# Using “Tender” X-ray Ambient Pressure X-Ray Photoelectron Spectroscopy as A Direct Probe of Solid-Liquid Interface

**DOI:** 10.1038/srep09788

**Published:** 2015-05-07

**Authors:** Stephanus Axnanda, Ethan J. Crumlin, Baohua Mao, Sana Rani, Rui Chang, Patrik G. Karlsson, Mårten O. M. Edwards, Måns Lundqvist, Robert Moberg, Phil Ross, Zahid Hussain, Zhi Liu

**Affiliations:** 1 Advanced Light Source, Lawrence Berkeley National Laboratory, Berkeley, California, 94720, United States; 2 State Key Laboratory of Functional Materials for Informatics, Shanghai Institute of Microsystem and Information Technology, Chinese Academy of Sciences, Shanghai 200050, People’s Republic of China; 3 VG Scienta, Uppsala SE-752 28, Sweden; 4 Materials Sciences Division, Lawrence Berkeley National Laboratory, Berkeley, California, 94720, United States; 5 School of Physical Science and Technology, ShanghaiTech University, Shanghai 200031, China

## Abstract

We report a new method to probe the solid-liquid interface through the use of a thin liquid layer on a solid surface. An ambient pressure XPS (AP-XPS) endstation that is capable of detecting high kinetic energy photoelectrons (7 keV) at a pressure up to 110 Torr has been constructed and commissioned. Additionally, we have deployed a “dip & pull” method to create a stable nanometers-thick aqueous electrolyte on platinum working electrode surface. Combining the newly constructed AP-XPS system, “dip & pull” approach, with a “tender” X-ray synchrotron source (2 keV–7 keV), we are able to access the interface between liquid and solid dense phases with photoelectrons and directly probe important phenomena occurring at the narrow solid-liquid interface region in an electrochemical system. Using this approach, we have performed electrochemical oxidation of the Pt electrode at an oxygen evolution reaction (OER) potential. Under this potential, we observe the formation of both Pt^2+^ and Pt^4+^ interfacial species on the Pt working electrode *in situ*. We believe this thin-film approach and the use of “tender” AP-XPS highlighted in this study is an innovative new approach to probe this key solid-liquid interface region of electrochemistry.

X-ray photoelectron spectroscopy (XPS) is one of the most powerful and versatile surface characterization techniques. It provides quantitative information about the elemental composition and chemical specificity^1^. Photon energies used for XPS, either by using laboratory-based or synchrotron radiation sources, typically are less than 2000 eV, and photoelectrons (PEs) generated by these soft X-ray sources have limited inelastic mean free path of only a few Angstrom (<20 Å) in solid materials^1^; thus conventional XPS is inherently a surface sensitive characterization technique. These attributes, however, impose constraints on conventional XPS measurements: they require ultra-high vacuum (UHV) conditions to avoid the electron scattering with gas molecules as well as the surface contaminations.

To apply XPS, also known as ESCA (Electron Spectroscopy for Chemical Analysis), to liquid and gas phases, Siegbahn *et al.* pioneered the methodology of ambient pressure XPS (AP-XPS) by using a laboratory-based X-ray source in near ambient pressure conditions^2^,^3^. Details describing the working principles of AP-XPS systems can be found in original reports and review articles^3^,^4^,^5^,^6^,^7^,^8^. In general, a sample is placed in a chamber with elevated pressure. A series of apertures are used to connect the elevated pressure chamber to the electron analyzer through a differential pumping section to reduce the PE effective travel length through the gas region and to protect the electron analyzer. Specially designed electron optics elements are typically incorporated in the differential pumping section to guide the PEs to go through apertures and increase the PE transmission.

During the past ten years, many developments in the use of AP-XPS have been made, including the use of synchrotron X-ray sources^5^,^6^,^7^,^8^,^9^,^10^. The introduction of high brightness synchrotron radiation sources and advanced AP-XPS instruments has led to tremendous progress in integrating realistic sample environments into surface science studies to address vacuum limitations. By pushing the operating pressure to higher values, these developments have transformed XPS from a surface science technique in vacuum to an important *in situ* tool for studies at solid-gas interfaces. Currently, AP-XPS is utilized in many important research fields such as heterogeneous catalysis, fuel cell, batteries, and environmental science^11^,^12^,^13^,^14^,^15^,^16^,^17^,^18^,^19^,^20^,^21^. Despite these achievements, some of the most important physical and chemical processes in nature, particularly in electrochemistry, take place at interfaces between solid-liquid phases. The lack of effective characterization tools, particularly *in situ* tools, has limited our understanding of the solid-liquid interface, an area also known as “the essence of electrochemistry”^22^,^23^,^24^. How to penetrate and characterize the interface between solid-liquid dense phases at the atomic and molecular level is still a challenge for the surface science and electrochemistry community.

Motivated by the success of surface science, pioneering works utilizing UHV surface techniques were carried out by researchers like Kolb, Hansen and *et al.* to extract atomic and molecular level information at the electrode-electrolyte interfaces^25^,^26^,^27^,^28^,^29^. Similarly, motivated by previously successful experiences at solid-gas interfaces, researchers started to explore new ways to study solid-liquid interface using AP-XPS^30^,^31^,^32^.

In this paper, we report a new method to probe the solid-liquid interface through the use of a thin liquid layer on a solid surface. We have constructed a new AP-XPS system equipped with a Scienta HiPP-2 electron analyzer and a three-electrode *in situ* electrochemistry apparatus. Combining this new system with a “tender” X-ray synchrotron source (an X-ray region 2 keV to <7 keV, between soft X-ray and hard X-ray), we are able to access the interface between liquid and solid dense phases with high energy PEs and directly probe important phenomena occurring at the narrow solid-liquid interface region in an electrochemical system.

We will discuss the advantages of using “tender” X-ray for probing the solid-liquid interface and provide a detailed description of the system performance. We then will introduce a “dip & pull” method to create a stable nanometers thick thin liquid film on a platinum electrode utilizing a customized three-electrode electrochemistry apparatus. Using this “solid-thin liquid film” system we show experimental evidence validating the thickness of this liquid (electrolyte) film and demonstrate this “solid-thin liquid film” system can be used for *operando* electrochemistry studies by probing Pt oxidation in 6 M KF electrolyte and discover the formation of Pt^2+^ and Pt^4+^ interfacial species during OER.

## Results

### “Tender” x-ray AP-XPS system and design principle

The ability to characterize the solid-liquid interface at the atomic and molecular level in realistic conditions while simultaneously obtaining detailed elemental and chemical composition information is the key to tackle some of the most fundamental and profound problems in nature as well as electrochemistry. In this regard, AP-XPS is a tool-of-choice if we can manipulate the surface sensitive nature of XPS and conduct the measurements at these realistic conditions. To do this, we need first to identify the optimal photon energy range to ensure that PEs are energetic enough to access the buried interface of interest while maintaining a decent sensitivity to the thin interface region. If hard X-ray photon is used, the resulting higher energy PEs can penetrate through thicker material. This is beneficial towards accessing the buried interface. Unfortunately, this large probing depth will reduce the relative contribution from the key interface region (typically ~1 nm thick). In addition, the higher the photon energy the lower the photo-ionization cross-section, thus it will further reduce the absolute photoemission signal from the interface region.

**Figure 1:**
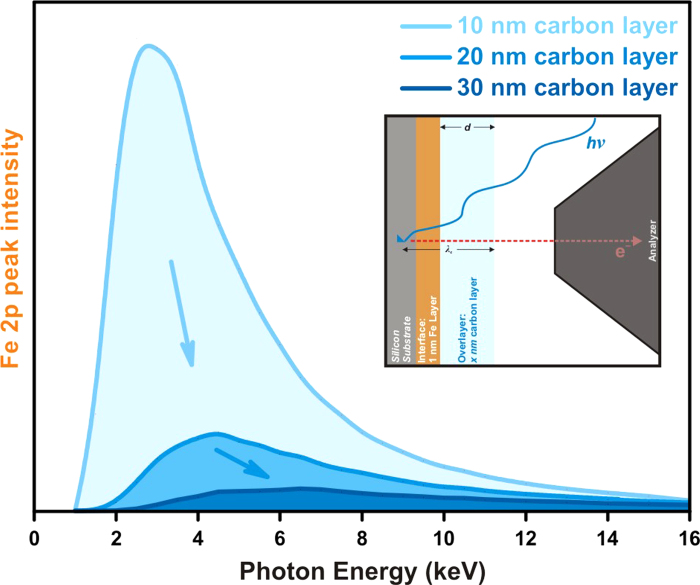
Integrated area of Fe *2p* for a 1 nm Fe interface layer as a function of photon energy buried under various thicknesses of carbon to illustrate the ideal photon energy region for studying interface phenomena in systems on the order of 10’s of nm in thickness. The inset is a representation of the electrolyte, electrode, and electrolyte-electrode interface layer used in the SESSA simulation used to generate the data for this plot.

In this section, we will demonstrate that “tender” X-ray (2 keV–7 keV) provides an optimal energy region for PEs to extract information from the interface region^33^. To illustrate this, we use SESSA software^34^,^35^ to simulate a model system consisting of a carbon over-layer of various thicknesses to mimic thin electrolyte layers. As shown in Fig. 1 (insert), we use a 1 nm thin layer of iron to represent the interface region (a typical thickness for electrical double layer) between the carbon layer and silicon substrate.

Using SESSA, we tabulate the intensity of the Fe *2p*_*3/2*_ core level signal at the buried interface as a function of incident photon energy for 10, 20, and 30-nm thick carbon over-layer in Fig. 1. For a 10 nm carbon over-layer, the Fe *2p*_*3/2*_ signal increases sharply at ~1 keV photon energy. It reaches its maxima at around 3 keV and decreases gradually as the photon energy increases. The sharp rise of intensity is due to the probing depth effect. At low photon energy (<1 keV), the PE inelastic mean free path (IMFP, ~1 nm) is much smaller than the thickness of an over-layer (either liquid or solid), therefore, little Fe *2p*_*3/2*_ signal can be detected. As the photon energy increases, the IMFP of the PE increases as well. We can start to detect the interface Fe *2p*_*3/2*_ signal when the PE’s IMFP approaches the thickness of the over-layer. As the photon energy increases further, the photo-ionization cross-section of Fe *2p*_*3/2*_ decreases significantly resulting in the decay of the Fe *2p*_*3/2*_ signal at the higher photon energies. Since the IMFP and photo-ionization cross-section are universally dependent upon electron kinetic energy and photon energy respectively for all core levels, we expect to see similar trends (Fig. 1) on other elements at the solid-liquid interface. As the thickness of carbon over-layer increases to 20 nm and 30 nm, both the onset photon energies and the optimal incident photon energies shift to higher energies due to the same IMFP and photo-ionization cross-section effects. In addition, the absolute Fe *2p*_*3/2*_ signal at the optimal photon energy also decreases significantly for the thicker film because of the diminishing photo-ionization cross-section (from the higher photon energy) and the attenuation from the thicker over-layer.

These SESSA XPS simulation results have two implications: 1) To probe the buried solid-liquid or solid-solid interface, the photon energy and model system used (over-layer thickness) need to be evaluated simultaneously. For the model system based on this nanometers thick thin-film over-layer, “tender” X-ray photons with energies between 2 keV–7 keV offer the best signal to noise ratio. 2) For model systems with more than ~30 nm over-layer, XPS may lose its advantage. Other techniques based on photon-in photon-out such as sum-frequency vibrational spectroscopy^36^ may become more attractive options. Furthermore, the electrolytes are typically low Z materials where the PE’s IMFPs are significantly larger than those of electrode materials with higher Z value. This makes it advantageous to probe the solid-liquid interface from the electrolyte side (the more PE transparent side). In short, this simulation demonstrates the tradeoffs between surface sensitive tools and bulk sensitive tools in probing the narrow interface region. Matching the right spectroscopy tool and photon energy range with specific model systems is crucial for many *in situ* experiment probing interfacial phenomena.

Following the aforementioned observation, we have built a new AP-XPS system to meet three requirements: 1) the ability to handle higher pressure than the current typical AP-XPS analyzer. It needs to be able to perform AP-XPS experiment at a pressure above 100 Torr (~1/7 atm), which is a pressure that will allow us to study most electrolytes at room temperature. 2) The ability to detect PEs efficiently with high kinetic energies up to 7 keV efficiently. 3) The ability to create and stabilize nanometers thick electrolytes on different electrode surfaces.

### Performance of “tender” X-ray AP-XPS endstation

**Figure 2:**
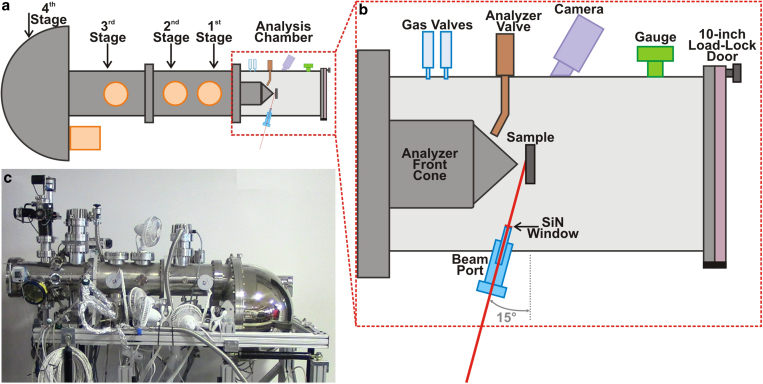
(**a**) Schematic top view of the “tender” X-ray AP-XPS analyzer and analysis chamber. (**b**) Detailed representation of the analysis chamber. (**c**) Photo of the actual “tender” X-Ray AP-XPS analyzer and the analysis chamber that is directly connected to the analyzer.

**Figure 3:**
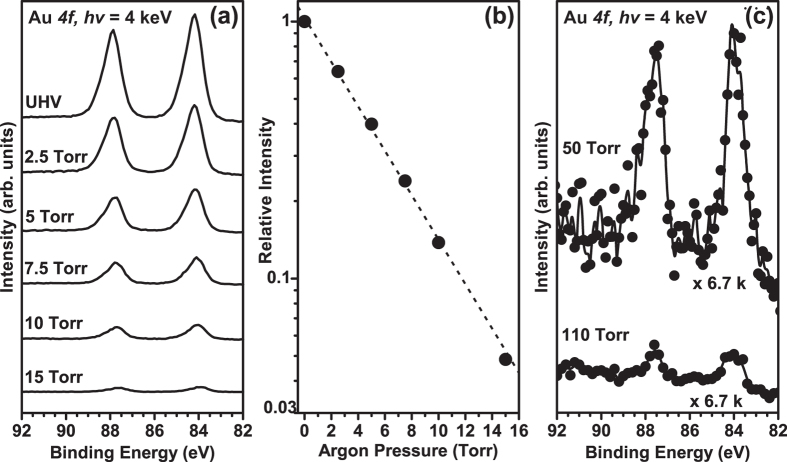
(**a**) Au *4f* spectra of Au foil in Ar gas at different pressures, (**b**) integrated intensity of the Au *4f* spectra relative to the vacuum condition as a function of Ar pressure, and (**c**) Au *4f* of Au foil in Ar gas at pressure ranges from 50 Torr to 110 Torr using a 0.1 mm diameter aperture cone.

A schematic and picture detailing the “tender” X-ray AP-XPS endstation is shown in Fig. 2, and more information can be found in the Supplementary Information S1 and S2. By monitoring the Au *4f* peak intensity of a Au sample in an argon atmosphere over a range of pressures while using a 0.5 mm cone and a photon energy of 4 keV, we are able to determine the performance of the analyzer to ensure it is capable of operating at pressures required. As shown in Fig. 3a, the intensity of Au *4f* signal at *P*_*Ar*_ = 2.5 Torr is reduced by 36% from the vacuum condition. This intensity decreases exponentially as the Ar gas pressure is increased to 15 Torr, culminating in 95% attenuation in intensity from the vacuum condition. Fig. 3b shows the relative intensity of the integrated Au *4f* signal as a function of argon pressure. This relative intensity data can be correlated to the PE effective path length is 0.51 mm for argon at 4 keV. Which nicely matches the ideal molecular flow path length of 0.53 mm (see detailed calculations in Supplementary Information S3). Thus the vacuum design of the HiPP-2 differential pumping system is efficient and can handle the aforementioned pressure requirements.

To further test the pressure range of this endstation, we have machined a 0.1 mm diameter front aperture using laser milling and attached it onto the apex of the cone with 0.8 mm opening. As shown in Fig. 3c, we can detect the Au 4f signal at a pressure at 50 Torr and as high as 110 Torr using this aperture. However, the intensity of Au 4f signal is greatly reduced at these pressures. In fact, the count rate at 110 Torr is 0.02% of the Au 4f measured at vacuum condition. Such a low count rate is only partially due to the increasing scattering from the gas at higher pressure. It is mainly limited by the current instrumentation limitations. (ie. beamline optics, X-ray shadowing of 0.1 mm aperture, see Supplementary Information S4), which can be resolved with future upgrades. For certain applications such as studying non-aqueous liquid organic electrolytes, it is important to enable efficient operation in this high gas pressure region.

### Creating the thin electrolyte film using “dip & pull” method on Pt

**Figure 4:**
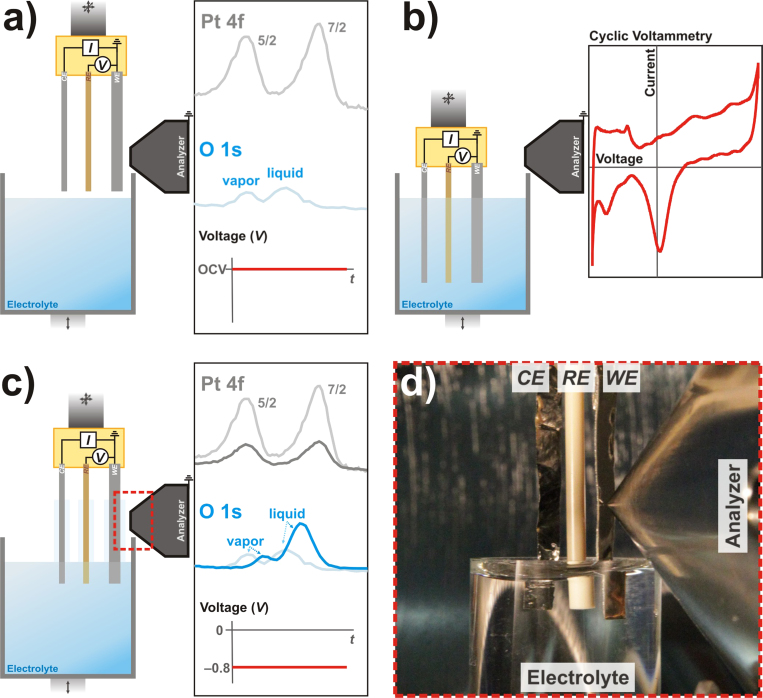
Schematic of three-electrode electrochemistry setup in the AP-XPS chamber. (**a**) Positions of electrodes before immersion and corresponding representative Pt *4f*, O 1*s* spectra and electrochemical profile. (**b**) Electrodes are immersed in the electrolyte, where any electrochemical treatment can be performed within the AP-XPS chamber. Shown is a representative Pt foil CV in 6 M KF aqueous electrolyte. (**c**) Electrodes are placed at the AP-XPS measurement position, and corresponding representative Pt *4f* and O *1s* of the partially removed electrodes are overlaying the representative vapor exposed electrode spectra (shown in Fig. 4a). (**d**) is an image of the 3-electrode apparatus that has been “dip & pulled” from the electrolyte in the beaker and placed into XPS position while ensuring all three electrodes are in contact with the electrolyte within the beaker.

To take advantage of this system’s capabilities and to facilitate electrochemical experiments, we developed a three-electrode electrochemistry apparatus capable of creating the solid-liquid interface for electrochemical applications. Fig. 4 shows the schematic detailing the three-electrode electrochemistry apparatus and the “dip & pull” procedure for creating a thin electrolyte layer on a working electrode (WE). More information of this method can be found in **Methods**.

Utilizing the three-electrode electrochemistry apparatus described above, we have performed experiments on a polycrystalline Pt WE. The 6 M KF solution is used as the electrolyte with a Pt polycrystalline foil as the counter electrode (CE). The Pt electrodes are flame annealed in air to remove the adventitious carbon. All the electrolyte containers are cleaned with Nochromix® cleaning solutions and sulfuric acid. The electrolyte also goes through a rigorous vacuum outgas procedure to remove dissolved gases in the solution. During the experiment, XPS spectra of Pt *4f*, O 1*s*, K 2*p*, and F 1*s* are collected under vacuum condition (<1 × 10^−3^ Torr) and water vapor pressures between 16 Torr and 20 Torr. All experiments were conducted at room temperature (298 K) using a 0.3 mm diameter aperture cone. Under these experimental conditions, the pressure in the analyzer region are always less than 4.0 × 10^−7^ Torr.

**Figure 5:**
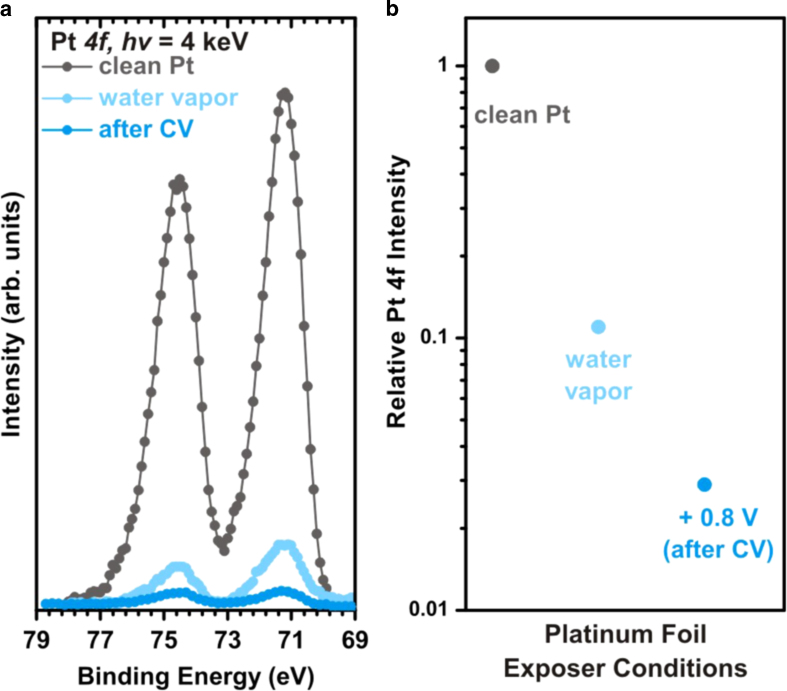
(**a**) Pt *4f* of clean Pt foil in 1 × 10^−3^ Torr (grey), under 20 Torr of water vapor pressure (light blue), and under 0.8 V potential with CV treatment (dark blue), (**b**) integrated intensity of the Pt *4f* spectra relative to the UHV condition at different treatment conditions.

Figure 5a shows the Pt *4f* spectra taken at different conditions. Firstly, the Pt *4f* spectrum of the clean Pt WE is taken under vacuum. After introducing 20 Torr of water vapor pressure (at 298 K) into the chamber, we collect the Pt *4f* again to determine the photoelectron attenuation from the water vapor. WE, CE and reference electrode (RE) are then dipped in the 6 M KF electrolyte container. We perform a cyclic voltammetry (CV) on the WE between –0.8 V and 0.8 V versus RE. After the Pt CV is stabilized, the Pt WE electrode is slowly pulled from the container and moved to the analysis position where the Pt *4f* spectrum is taken. During this process, we ensure that part of each electrode remains immersed in the electrolyte. Therefore, the electrolyte thin film created on the WE can be electrically connected to the bulk electrolyte. As shown in Fig. 5a, the intensity of this Pt *4f* decreases significantly after the electrode is subjected to the CV treatment in the electrolyte. The dark blue line shows the Pt *4f* spectrum after the electrode surface is exposed to CV treatment and “dip & pull” procedure. Compared to that of clean Pt foil in vacuum condition, Pt *4f* intensities upon water vapor exposure and CV treatment are attenuated to 11.0% and 2.9% respectively as shown in Fig. 5b. This indicates that the formation of a thin electrolyte layer on the Pt electrode surface after the “dip & pull” procedure.

## Discussion

### Verification of the thin electrolyte film thickness

Using the Pt *4f* intensity attenuation shown in Fig. 5b, we can now calculate the thickness of the electrolyte layer. By assuming that the electrolyte covering the WE surface is in the form of a thin layer after the electrochemical treatment, the Pt *4f* intensity of WE, *I*_*EC*_, escaping through an over-layer of thin electrolyte film is^37^.


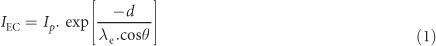


where *I*_*p*_ is the Pt intensity from the electrode exposed to water vapor only; *d* is the thickness of the surface electrolyte layer; and electron escape depth is *λ*_*e*_. *θ* is the angle between the escaping electron with the sample surface normal. We use the data from Emfietzoglou *et al.*^38^. to determine the electron escape depth, *λ*_*e*_, in equation (1). For example, at 4 keV photon energy (3.92 keV kinetic energy for Pt *4f* photoelectron), *λ*_*e*_ is 10 nm for water (to represent our electrolyte solution). For this system, the collected photoelectrons are traveling perpendicular to the Pt surface, hence *θ* is 0°. Using the aforementioned Pt *4f* intensities for water vapor (11.0%) and immersed after CV (2.9%), we can now determine the thickness of the electrolyte layer using Equation (1), 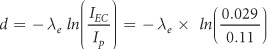. For a 10 nm electron escape length, the thickness of the thin electrolyte is ~13 nm after the electrochemical treatment. We also find this thin film is stable during the experiment as long as the water vapor pressure is kept constant.

Several similar “dip & pull” experiments are performed. The electrolyte thickness on Pt WE is found to be in the range of 10 nm to 30 nm. Beside the water vapor pressure dependence, we also observed correlations between the electrolyte film thickness and electrochemical treatments performed on the Pt WE prior to the pulling. Such correlations can be due to the fact that the Pt surface is modified under the CV treatment and its hydrophilicity is changed under different electrochemical treatments. We plan to address the origin of this observation in the future study. Nevertheless, the interaction of the halogen ions with Pt surfaces has been extensively studied^28^. It is known that the adsorption of halide ions on Pt surface are influenced by an applied potential. The adsorption of these ions will affect the balance of electrostatic and covalent interactions at metal/electrolyte interfaces,^23^,^25^,^26^,^27^,^28^,^29^ which can lead to the change of the wetting behavior of the Pt surface. With AP-XPS, we can directly study these interactions and their origins.

### The conductive nature of the thin electrolyte film

**Figure 6:**
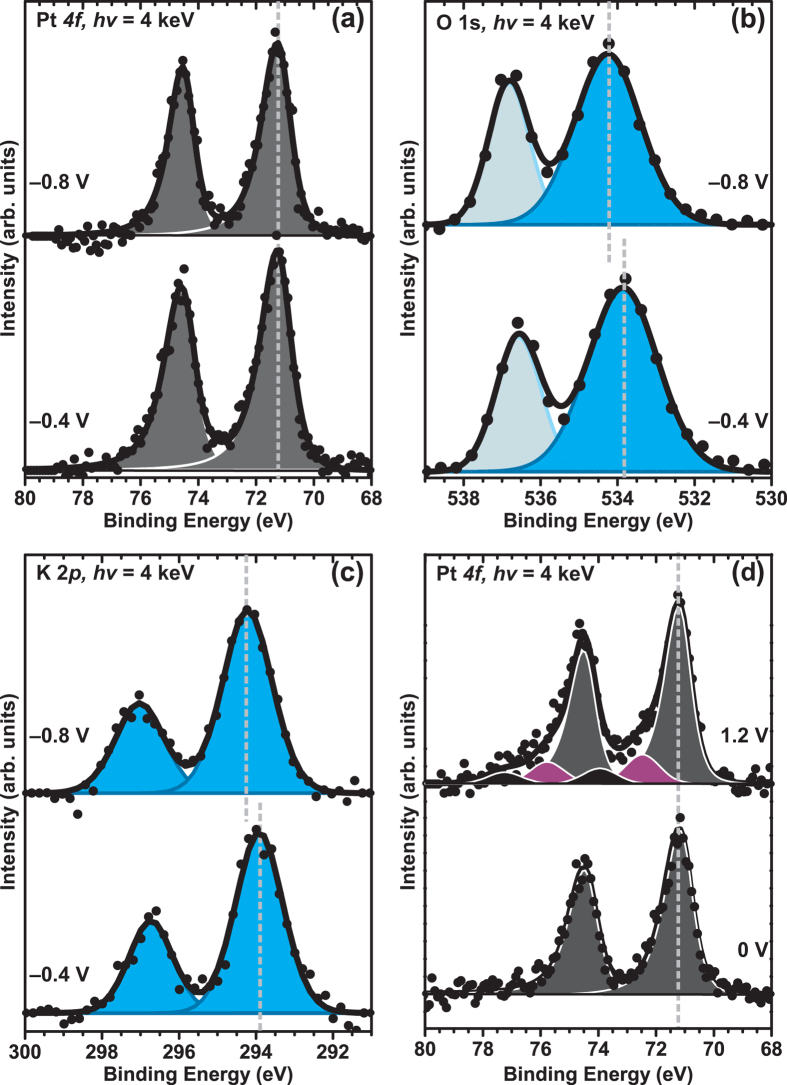
(**a**) Pt *4f*, (b) O *1s*, and (**c**) K *2p* of Pt foil after dipped in 6 M KF after cyclic voltammetry between −0.8 V and 0.8 V is applied, followed by holding the potential at −0.8 V and −0.4 V, and (**d**) Pt *4f* with potential holding at 0 V and 1.2 V during the XPS measurement. All of the potentials are reported with respect to Ag/AgCl reference electrode.

Once the formation of the stable thin electrolyte layer on the Pt WE surface using the “dip & pull” technique is established, we need to determine whether this nanometer thick layer is conductive and can act as an active functional electrolyte layer. For this purpose, we compared the XPS peaks of different elements from the Pt WE (Pt *4f*) and electrolyte layer (O *1s* and K *2p*) by applying different electrochemical potentials to this thin film formed. In this experiment, the Pt WE is immersed into 6 M KF followed by the same CV from −0.8 V to 0.8 V (vs Ag/AgCl) potential window, and is concluded by holding the potential at certain value. Then the three electrodes are partially pulled out of the electrolyte container and moved to the analysis position (as shown in Fig. 4a-4c).We expect that XPS core level peaks from the electrolyte elements to show certain binding energy (*BE*) shifts following the changes in the applied potentials if the thin film is indeed conductive and connected to the bulk electrolyte. Results taken at −0.8 V and −0.4 V holding potentials from this experiment are shown in Fig. 6a–6c.

In Fig. 6a, no *BE* shift is observed on Pt *4f* while comparing spectra taken at −0.8 V and −0.4 V. This observation is consistent with the fact that the Pt WE is grounded along with the XPS analyzer. No electrical potential shift, i.e. *BE* shift from Pt *4f*, is expected. From the electrolyte, the O *1s* spectrum (Fig. 6b) shows 2 main peaks, *BE*s at ~534 eV and ~537 eV representing the electrolyte liquid phase from the thin film, and water vapor phase, respectively^39^. Comparing the *BE* positions of bulk liquid peaks taken at −0.8 V and −0.4 V, we find the O *1s BE* shifts from 534.3 eV at −0.8 V holding potential to 533.9 eV at −0.4 V holding potential. This 0.4 eV *BE* shift matches the electrochemical potential shift of the WE with respect to the Ag/AgCl reference electrode. Because the WE is grounded to the analyzer, we expect such an applied potential change be fully reflected into the *BE* shift of the electrolyte. The fact that the observed electrolyte (O *1s*) *BE* shift matches exactly the potential difference is the direct evidence of the conductive nature of the thin electrolyte film formed. Otherwise, the electrolyte liquid phase peak at ~534 eV would not change with applied potential if the electrolyte film was either non-conductive or is not connected to the bulk electrolyte container. This shows that the thin electrolyte film can indeed preserve the electrochemical potential difference across the solid-liquid interface.

In addition, if the film was discontinuous or mainly comprised of isolated patches/droplets, these patches will not be in electrical contact with the bulk electrolyte and they will take the Pt WE potential. Then we would expect to see no *BE* change in electrolyte O *1s* peak with different applied voltages or we would see split peaks if there is a mixture of isolated patches and continuous film. As we only see one single electrolyte O *1s* peak shifting with applied potentials, this is clear proof that that the thin film formed is continuous with few if any isolated patches and connected film to the bulk electrolyte. In Fig. 6c, we also show K *2p* spectra in both conditions. The presence of K *2p* confirms that an electrolyte layer is actually formed on the surface since the electrolyte is comprised of KF (as opposed to just being a water film). The K *2p* spectrums have two main peaks due to the spin-orbit splitting (K *2p*_*3/2*_ and K *2p*_*1/2*_) and show similar trend in *BE* shift as the liquid phase O *1s* spectra. Both K *2p* and O *1s* electrolyte peaks shift towards lower *BE* values as the potential applied is changed to a more positive value. Such electrochemical shift of an element’s *BE* due to electrostatic potential has been observed and is assigned due to the shift of the electronic states of the respective element^16^,^40^,^41^,^42^. In many previous *ex situ* XPS studies of immersed electrodes, researchers have observed such systematic shifts of the proposed electrochemical double layer species with electrode potential^26^,^40^,^43^. Herein, we can use a photoelectron transparent and functioning thin electrolyte film to investigate the electrochemical double layer and other electrochemical phenomena originated at the solid-liquid interface *in situ*.

### *In situ* Pt electrode oxidation using the thin electrolyte film

To further demonstrate the capability of this thin electrolyte formed, we conduct an *in situ* Pt oxidation of the Pt WE. We want to show that the film can be used as a functional electrolyte. Following the previously described “dip & pull” procedure, the Pt WE is immersed into 6 M KF followed by CV from −0.8 V to 0.8 V (vs Ag/AgCl). Once the Pt CV is stabilized, the Pt electrode is pulled from the electrolyte and moved to the analysis position (position as shown in Fig. 4c) while holding the potential at −0.8 V. After the electrolyte film is formed, we then change the applied voltage to 0.0 V and 1.2 V *while keeping the Pt WE electrode at the analysis position*.

We show Pt 4f spectra in Fig. 6d. At 0.0 V holding potential, Pt 4f shows a single metallic state represented by the fitting of Pt *4f*_*7/2*_ using one peak only that is located at *BE* of 71.2 eV (shown in grey). At 0.0 V, the Pt is metallic. Upon changing the holding potential to 1.2 V, new features on the higher binding energy side of the Pt metallic peaks appear. To fit the Pt *4f*_*7/2*_ spectrum at 1.2 V, two additional peaks at *BE* of 72.6 eV (shown in purple color) and 74.1 eV (shown in black color), which are 1.4 eV and 2.9 eV higher than that of the metallic Pt peak, were required.

With a measured pH value of 8.5 for 6 M KF electrolyte solution (obtained using an Oakton/pH 510 series pH meter), a holding potential of 1.2 V with respect to Ag/AgCl on Pt in 6 M KF solution is located well into the OER region. From the Pourbaix diagram^44^, the oxidation of Pt is expected in this OER region and PtO_2_ is thermodynamically stable species at this potential. However, the observed Pt *4f*_*7/2*_ peaks have a major component at 72.6 eV as well as a smaller peak at 74.1 eV. The 1.4 eV core level shift (CLS) value is much less than reported values for PtO_2_/Pt^4+^^45^,^46^,^47^,^48^,^49^,^50^,^51^,^52^,^53^,^54^,^55^,^56^. The PtO_2_/Pt^4+^ CLSs observed in all these studies are larger than 2.5 eV, most of them have a CLS value close to 3.5 eV. Therefore, we can rule out PtO_2_/Pt^4+^ as the exclusive interfacial species observed in this study. It has also been reported that an additional Pt *4f* peak with 1.4 eV CLS sometimes is observed along with the main PtO_2_ oxide peak at higher *BE*^45^,^56^. These two components are assigned to Pt species with +2 and +4 formal valence state, respectively. In one recent publication, Arrigo *et al.* report a CLS of 1.3 eV in their *in situ* OER study during the gas phase water electrolysis on Pt electrode^57^. They assigned this component to a divalent state of a hydrated Pt^2+^ oxide. This is consistent with the observation here. Following the aforementioned literature values, we also assign the observed 72.6 eV Pt 4*f* peak to be the oxidation state of Pt^+2^ and 74.1 eV Pt 4*f* peak to be the oxidation state of Pt^+4^. Thus we can conclude that the Pt interfacial species observed at this OER potential are a mixture of both Pt divalent state and Pt teravalent state, which is different from the pure teravalent state suggested by the Pourbaix diagram at this pH range. We would like to point out that the signal to noise ratio of the Pt 4*f* spectrum is less than ideal in this first experiment. In addition, we do not identify the mixture of both Pt divalent state and Pt teravalent state as the active Pt phase in OER reaction. Data of better quality are needed for more qualitative analysis of the Pt oxidation state at OER conditions. The upgrade of the beamline 9.3.1 is currently underway to improve the focusing of the X-ray. The detailed discussion of Pt oxidation states and structures at OER region is beyond the scope of this report and will be reported elsewhere.

Nevertheless, by changing the applied potential on the thin electrolyte film *in situ*, we have observed clear oxidation state changes of the Pt WE. We can conclude that the thin electrolyte layer formed on the surface of Pt is an “active” and functional layer. The electrical potential across the electrode-electrolyte interface can be maintained. Therefore, this thin electrolyte film based method allows us to perform *operando* studies on electrochemical systems using the AP-XPS system. Furthermore, these preliminary results show that the chemical states of Pt electrode during the OER include a divalent state, which can be the result of a kinetics driven product. This observation highlights the importance of *in situ/operando* research.

Yet, such a thin film approach has its limitations that should be treated with caution. Due to the thickness limitation of the thin film, the ion mass transport along the film direction is limited. This mass transport limitation can, in principle, affect the ion concentrations of the solution, such as pH value, at different vertical position. However, the ability to measure the relative concentration of different ion species and water using XPS at given positions and voltages allows us to identify these mass transport related issues. We are currently evaluating these phenomena.

In summary, we have constructed a new “tender” X-ray AP-XPS endstation based on the Scienta Hipp-2 analyzer. This endstation can detect high-energy photoelectrons up to 7 keV and extends the current pressure range of AP-XPS experiment up to 110 Torr. In addition, a “dip & pull” method is deployed and utilized to create a stable nanometers thick aqueous electrolyte on the Pt WE surface. We have demonstrated that the thin electrolyte layer (10–30 nm thick) created is continuous and can carry the electrical potential of the bulk electrolyte. Using this film, we have performed *in situ* electrochemical oxidation of Pt WE in 6 M KF electrolyte. The oxidation states of interfacial Pt species discovered are a mixture of Pt^2+^ and Pt^4+^ at the OER potential of 1.2 V (with Ag/AgCl RE). We believe that this thin film approach is an important step for studying electrochemical systems using XPS. It successfully enables us to take advantage of the penetrating power of “tender” X-ray and to perform *in situ/operando* AP-XPS research at the narrow electrolyte-electrode interface with optimal efficiency. Protected by the thin electrolyte layer, the atomic and molecular structure of the solid-liquid interface layer between the electrolyte and electrode can be preserved and adjusted during the XPS measurements. This approach opens a transparent window for many powerful *in situ* characterization tools like AP-XPS to probe the key region of electrochemistry. Last but not the least, the simplicity of this approach makes it very adaptable for other experimental setups.

## Methods

### “Dip & pull” procedure

In Fig. 4a (right hand side), the working electrode (WE, Pt foil) is placed closest to the analyzer cone. The two remaining electrodes consist of a Ag/AgCl reference electrode (RE) and a Pt foil counter electrode (CE). All three electrodes are mounted into a PEEK electrode housing that is attached to a multi-axis manipulator. Electrical feed-through within the manipulator connect the WE, RE, and CE to an external potenstiostat to perform *operando* electrochemistry measurements. During the operation, the WE and the analyzer front cone are grounded. The electrolyte of choice is placed in a glass container that is mounted on a linear translator. This linear translator can be changed to a three-axis manipulator for additional degrees of freedom. Hence, both electrode and electrolyte position with respect to the analyzer can be adjusted to increase experimental flexibility. When aqueous electrolyte is used, the water vapor pressure in the main chamber is controlled up to 20 Torr through a UHV leak valve connected to a heated water reservoir. At the equilibrium condition, this allows the electrolyte level in the glass electrolyte container to remain constant during the experiment. For this setup, both the WE and the CE are exchangeable. Samples that meet certain geometrical requirements can be used as the WE or CE. Thin films of metals and metal oxides on various substrates such as silicon wafers, glass, and HOPG have been used.

In Fig. 4a, the Pt WE is placed in front of the analyzer cone where spectra of the bare electrode in a vacuum or gas environment can be collected. Representative Pt *4f* and O *1s* XPS spectra from the Pt WE under water vapor are schematically depicted in Fig. 4a as well. This is a standard AP-XPS gas-solid interface experimental configuration. In Fig. 4b, the electrodes are moved away from the analyzer and fully immersed into the electrolyte. At this position, we can also initiate and perform the desired electrochemical pretreatments on the Pt electrode and carry out *in situ* measurements, such as cyclic voltammetry (CV) and others as needed.

To create a solid-liquid interface (WE-electrolyte), all three electrodes are immersed into electrolyte. Then they are slowly extracted from the bulk electrolyte solution by either rising the three electrodes or lowering the electrolyte container. Following this procedure, a thin layer of liquid electrolyte film is formed on the Pt electrode. By moving the three-electrodes and electrolyte container accordingly, this thin film can be positioned at the focal point of the analyzer and studied using XPS (Fig. 4c). Depending on the position of the electrolyte container, we can study the electrolyte film at different positions with respect to the bulk electrolyte surface where the meniscus thickness can be different.

During the measurement, all three electrodes can be kept in contact with the bulk electrolyte. Therefore, the electrolyte film on the WE stays in contact with bulk electrolyte. It allows the simultaneous collection of spectra from the WE (Pt *4f*), electrolyte (O *1s*) and water vapor gas phase (O *1s*) at given holding potentials, which is schematically represented in Fig. 4c. Fig. 4d is an image of the 3-electrode apparatus that has been “dip & pulled” from the electrolyte in the beaker and placed into XPS position while ensuring all three electrodes are in contact with the electrolyte within the beaker.

More images and a video of the “dip & pull” method can be found in the Supplementary Information S5

### AP-XPS measurement

AP-XPS measurements were performed at Beamline 9.3.1 of the Advanced Light Source of the Lawrence Berkeley National Laboratory (Berkeley, USA). Beamline 9.3.1 is a bend magnet beamline with a energy range of 2.3–5.2 keV. The minimal spot size at the beamline is 0.7 mm (v) x 1.0 mm (h).

## Additional Information

**How to cite this article**: Axnanda, S. *et al*. Using "Tender" X-ray Ambient Pressure X-Ray Photoelectron Spectroscopy as A Direct Probe of Solid-Liquid Interface. *Sci. Rep.* 5, 9788; doi: 10.1038/srep09788 (2015).

## Supplementary Material

Supplementary Information

Supplementary Movie
