# Supplementary material for: Using “Tender” X-ray Ambient Pressure X-Ray Photoelectron Spectroscopy as A Direct Probe of Solid-Liquid Interface
Source: Sci Rep. 2015 May 7;5:9788. doi: 10.1038/srep09788 (PMC4650780; doi:10.1038/srep09788)
Supplement: Supplementary Information [file srep09788-s1.pdf]

Supplementary Information

Using "Tender" X-ray Ambient Pressure X-Ray Photoelectron Spectroscopy as A Direct Probe of Solid-Liquid Interface

Stephanus Axnanda[1], *Ethan J. Crumlin*[1], *Baohua Mao*[1,2], *Sana Rani*[1], *Rui Chang*[1,2], *Patrik G. Karlsson*[3], *Mårten O. M. Edwards*[3], *Måns Lundqvist*[3], *Robert Moberg*[3], *Phil Ross*,[4,*], *Zahid Hussain*[1], *Zhi Liu*[1,2,5*]

1. Advanced Light Source, Lawrence Berkeley National Laboratory, Berkeley, California, 94720, United States
2. State Key Laboratory of Functional Materials for Informatics, Shanghai Institute of Microsystem and Information Technology, Chinese Academy of Sciences, Shanghai 200050, People's Republic of China
3. VG Scienta, Uppsala SE-752 28, Sweden
4. Materials Sciences Division, Lawrence Berkeley National Laboratory, Berkeley, California, 94720, United States
5. School of Physical Science and Technology, ShanghaiTech University, Shanghai 200031 , China

*e-mail: zliu2@lbl.gov, pnross@lbl.gov

S1: "Tender" AP-XPS Endstation: It can be divided into two main parts: R4000 HiPP-2 analyzer (Fig. 2a) and the analysis chamber (Fig. 2b). The analysis chamber and the electron energy analyzer are connected through a 10-inch conflate flange. The differentially pump analyzer pre-lens system is housed in a Mu-metal chamber and separated from the analysis chamber through an aperture at the apex of the front cone. To detect high kinetic energy electrons at high pressure and maintain a stable operation, the HiPP-2 analyzer uses a shaped mesh as one of the lens elements in the pre-lens. The detailed description of this unique design and how we achieved both high-energy resolution and high electron transmission with R4000 HiPP-2 analyzer can be found in the Supplementary Information S2.

Fig. 2b shows the top view of the analysis chamber. To accommodate different *in situ* configurations, a large analysis chamber is selected. The chamber has an inner diameter of 20-cm and a length of 48-cm. A specially designed valve is installed which can quickly seal off the analyzer from the analysis chamber. Thus facilitating rapid venting and convenient access for experimentation setup through the large 10-inch load-lock door located at the front of the analysis chamber. Individual gas or gas mixtures can be introduced into the chamber through two UHV leak valves. The beam port that connects the system to the beamline has a 15° incident angle with respect to the sample surface. A 500-nm thick Si_3N_4 window (96% transmission at photon energy of 3 keV) to isolate the analysis chamber and the beamline at the end of beam deliver port.

Two manipulator ports (not shown) are located directly above and below the analyzer cone where two manipulator systems can be utilized to move the experimental setups simultaneously. The top sample manipulator is equipped with a four-axis motor system for computer control of sample motion by step motors with submicron precision. For normal gas-solid system operation, we can use modified Thermionics STLC platens as described in a previous report.[1] We can mount a ceramic coated button heater (HeatWave) for heating, a specially designed Peltier carrier for cooling, or an electrochemical platen for electrochemical experiment.[1] A special three-electrode electrochemistry apparatus is also developed for creating a stable thin layer of electrolyte on different electrode surfaces. The three-electrode electrochemistry apparatus is constructed out of PEEK material, which houses all three electrodes for electrochemical

experiments (for this report the three electrodes are: working – Pt foil, counter – Pt foil, and the reference – Ag/AgCl). The electrodes are electrically connected to a potentiostat located outside the chamber.

As shown in Fig. 2b, the R4000 HiPP-2 analyzer is connected to the main chamber through a front cone made of titanium with a small aperture opening at the apex. This cone is removable and interchangeable. For this system, the front Ti cone has a half angle of 45-degrees. Cones with aperture diameters of 0.1 mm, 0.3 mm, and 0.5 mm have been used and tested. Using Ar gas with the 0.3-mm diameter aperture cone, the ratio between the pressure at the analyzer (located at the 4th stage) and the pressure of the analysis chamber is 2 x 10^{-8} Torr. A Varian 250 l/s turbo-molecular pump is used at each of the four pumping stages. Therefore, the differential pumping system is sufficient to maintain the high vacuum condition (< 10^{-6} Torr) required at the analyzer while the main chamber is back filled with elevated gas pressure.

Using the nomenclature of Ogletree et al.,[1,2] the maximum pressure (P_{MAX}) for an AP-XPS system is defined as the effective path length for PEs at this pressure to the analyzer is twice of the PE's IMFP. Therefore, $\lambda_e = kT/P_{MAX}\sigma_e$, where k, T, and σ_e are the Boltzmann constant, temperature, and the electron cross-section, respectively. Under these conditions the measured intensity is roughly 13% of the intensity measured in vacuum. While this value is arbitrary, it serves as a good comparison between systems. It is also known that the sample must be placed a distance at least twice the cone radius (R) away from the aperture to ensure the pressure at the sample is at least 95% the pressure inside the experimental chamber.[1] At this minimum distance, the maximum pressure can be correlated to the cone size following this expression $P_{MAX} \approx kT/R\sigma_e$. The electron scattering cross-section depends on PE kinetic energy (thus also the photon energy) and the gas species. For a 4 keV photon energy, the σ_e of Au $4f$ PE through Ar gas at room temperature is 0.9 x 10^{-16} cm^2,[3] which leads to a $P_{MAX} \approx 23$ Torr when R = 0.15 mm. As a comparison, if a 400 eV soft X-ray photon is used, the value of P_{MAX} is reduced to 9 Torr (σ_e= 2.3 x 10^{-16} cm^2). This is much smaller than the P_{MAX} value that can be achieved with a photon energy of 4 keV. Therefore, higher photon energy as well as smaller aperture size can be used to achieve the higher P_{MAX} in AP-XPS.

S2: R4000 HiPP-2 Analyzer for high energy PE detection

To detect high kinetic energy electrons at high pressure and maintain a stable operation, the HiPP-2 analyzer uses a shaped mesh as one of the lens elements in the pre-lens, and at high kinetic energy operation this mesh is on ground potential. The receiving lens are typical R4000 XPS lens, similar to what is described by Mårtensson et al.[4] As will be further discussed below, using a shaped mesh lens element in a lens designed for collecting high energy electrons from a high pressure environment has several advantages: A large angular range can be collected without having extreme spherical aberrations.[5] The electron optical properties introduced by the mesh electrode allows for a relatively long distance between the sample and the first section of strong electrical field, which is beneficial for suppressing corona discharge. Adopting the shaped mesh lens also allows the first cone to be made sharper, which improves the access to the sample position without scarifying too much performance. Furthermore, it is possible to obtain focusing and retardation simultaneously. This is very important for the operation at high energies because this implies that the lens voltages can be kept relatively low.

On the other hand, there are also disadvantages for using mesh electrodes. For example, having an electric field at the shaped mesh interface will inevitably produce a micro lens effect for each individual transparent part of the mesh. This effect cannot be removed by changing the thickness, or the wire density, of the mesh. Fortunately, improvements of both manufacturing technique and theoretical modeling[6] of the shaped mesh electrodes have resulted in good agreement between theory and experiment. We can predict and estimate many of the side effects with high accuracy. By improving the details of the theory, taking micro lens effects into account, the overall analyzer performance can be analyzed and found to be slightly reduced. The total effect of the micro lens effect is very similar to what happens when the size of the photon spot is increased. Hence, the most important trade-off in using a lens with a shaped mesh as one of the lens elements is that the most extreme angular resolution cannot be achieved and extreme spatial resolution of an imaging lens mode is not possible.

Fig. S1 shows a model cross sectional view of the HiPP-2 pre-lens. The horizontal axis is the optical axis of the lens, and is denoted the z-axis. The vertical axis is along the hemispherical energy analyzer slit plane, and is denoted the x-axis. The y-axis is in the direction out of the plane y = 0. Active lens elements are rotational symmetric around the z-axis, and the y=0 plane is a mirror plane for the slits. A simulated rectangular emitting sample of 0.4 mm and 0.2 mm in

the x- and y-directions, respectively, is centered at the position (x,z) = (0,0). For the simulated trajectories the lens is set for 4525 eV initial kinetic energy and pass energy 200 eV, a retardation ratio of 22.625. The colored background represents the kinetic energy of electrons with initial energy 4525 eV. On the sample side of the shaped mesh the white color represents 4525 eV, and at the pre-lens exit slit position the blue color represents 1530 eV, which is about one third of the kinetic energy initial value. On the pre-lens exit slit side of the shaped mesh three kinetic energy contours are inserted. This design allows for not applying any electrical field at the very front of the first cone where the pressure would be the largest to avoid the potential for corona discharge. Furthermore, the kinetic energy is decreased in a step-wise manner to ensure moderate internal lens voltage differences. It is important to point out that the exit slits of pre-lens are not held at ground potential. Therefore, the receiving lens internally works at a lowered retardation ratio, which lowers the internal lens voltage differences also in this lens.

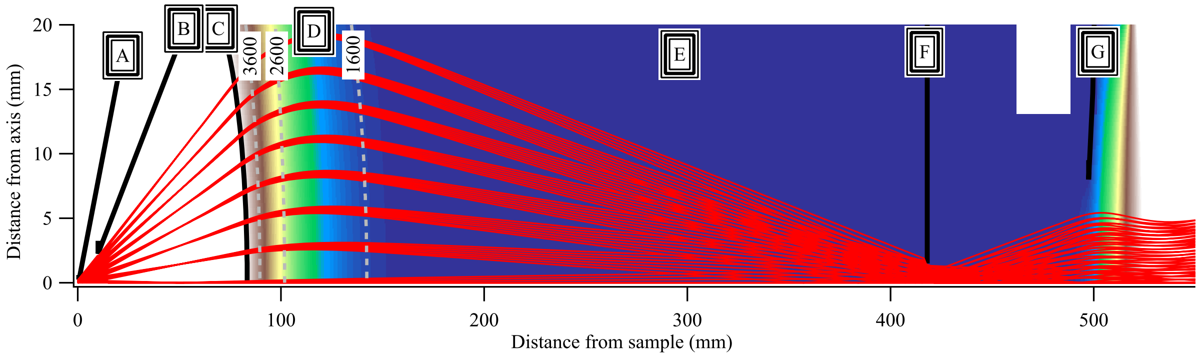

Figure S1. Front cone, always grounded. B. Second cone, part of L1p lens element, grounded for high energy operation. C. Shaped mesh electrode, part of the L1p lens element, grounded for high energy operation. D. Retarding lens elements L2p and L3p (geometry not shown). Kinetic energy contours for initial energy 4525 eV inserted. E. Quadrupole deflector (geometry not shown). F. Pre-lens exit slit, on L3p retarding potential. G. Front of receiving lens electrically connected to the L3p retarding potential.

With the shaped mesh put on ground potential the geometrical maximum acceptance angle for the shaped mesh is 30 degrees, i.e. 15 degrees from optical axis. Although it is possible to push the steep angles in the slit plane to reach the detector, we cannot find this to be beneficial for high count rate[4] operation at high kinetic energies under the high pressure sample environment. In Fig. S1, the largest take off angle is modeled to be 11 degrees from the optical axis. For

differential pumping purposes the geometrical dimensions of the second cone limit the acceptance angle to 26 degrees. The spatial magnification, angular magnification, and retardation ratio are correlated and bounded.[7] Therefore, the pre-lens is set to create an output, which the receiving lens can efficiently transmit to the hemispherical analyzer. In order to keep the energy resolution, as set by the dimension of the lens exit slit (analyzer entrance slit) in the energy dispersive direction, the hemispherical energy analyzer needs to limit the beam divergence in the energy dispersive direction. This is realized by combining the lens exit slit with a lens aperture slit.[8] As we know, increasing the spatial magnification reduces the beam divergence and lowering the kinetic energy increases the beam divergence. Additionally, spherical aberrations affect the quality of the image.[9] A good compromise has been found for a total spatial magnification of 6 times. For the most commonly used slits and operation regime the contributions to the transmitted solid angle from take-off angles larger than 11 degrees from the optical axis is very limited. Thus there is not much to gain by increasing the acceptance angle for high energy operation. However, using lower retardation ratios combined with lower kinetic energies, it is possible to efficiently transmit steeper angles. Collection of angles up to the acceptance of the first cone (up to 22.5 degrees) can be realized by putting the L1p on relative high positive voltage. Further discussion regarding this operational mode is out of the scope for this paper.

In summary, we find that the use of a shaped mesh lens in the HiPP-2's prelens enable us to detect high kinetic energy electrons at high pressure and maintain a stable operation.

S3: PE effective path length

d_{eff}, following $\ln\left(\frac{I}{I_0}\right) = -d_{eff}\sigma_e P_0/kT$, where σ_e= 0.9 x 10^{-16} cm^2 for argon at 4 keV. From the intensity data, we find that the PE effective path length is 0.51 mm. This value can be compared with the calculated effective path length under condition of the ideal molecular flow for which, $d_{eff} = \frac{1}{2}(\sqrt{1+z^2} - z)$, where z is the position of sample in units of R. For z = −0.5 mm, d_{eff} = 0.53 mm. This is within the uncertainty of the experimentally determined value of d_{eff} = 0.51 mm. The differential pumping of the gas at this pressure range can still be simulated by ideal molecular flow.

S4: Instrumentation limitations and future updates

Firstly, The large X-ray beam size (1 mm x 2 mm), limited by the beamline optics during the time of measurement, leads to a much larger beam spot on the sample than the diameter of the aperture and contributes to this low count rate. Secondly, the sample cannot move closer than 0.2 mm to the aperture due to the X-ray shadowing of this homemade aperture: We have machined a 0.1 mm diameter front aperture using laser milling on a 1mm stainless steel disk and attached it onto the apex of the cone with 0.8 mm opening. This 1mm disk will shadow the Xray beam and preventing it from illuminating the region close to the aperture. The resulting longer than optimal sample to aperture distance also significantly increases the gas attenuation and deceases the count rate. Therefore, this count rate issue can be improved significantly with a more focused X-ray spot and a better designed aperture (sharper front cone and smaller sample distance). For example, a 50-micron (vertical) x 100-micron (horizontal) spot size can comfortably be achieved with current synchrotron beamline technology. This will lead to a reduction of 400 in spot size, which will match well with the aperture size. For certain applications such as studying non-aqueous liquid organic electrolytes, it would be important to carry out these upgrades to reach such a high pressures.

S5: Images of "dip & pull" method:

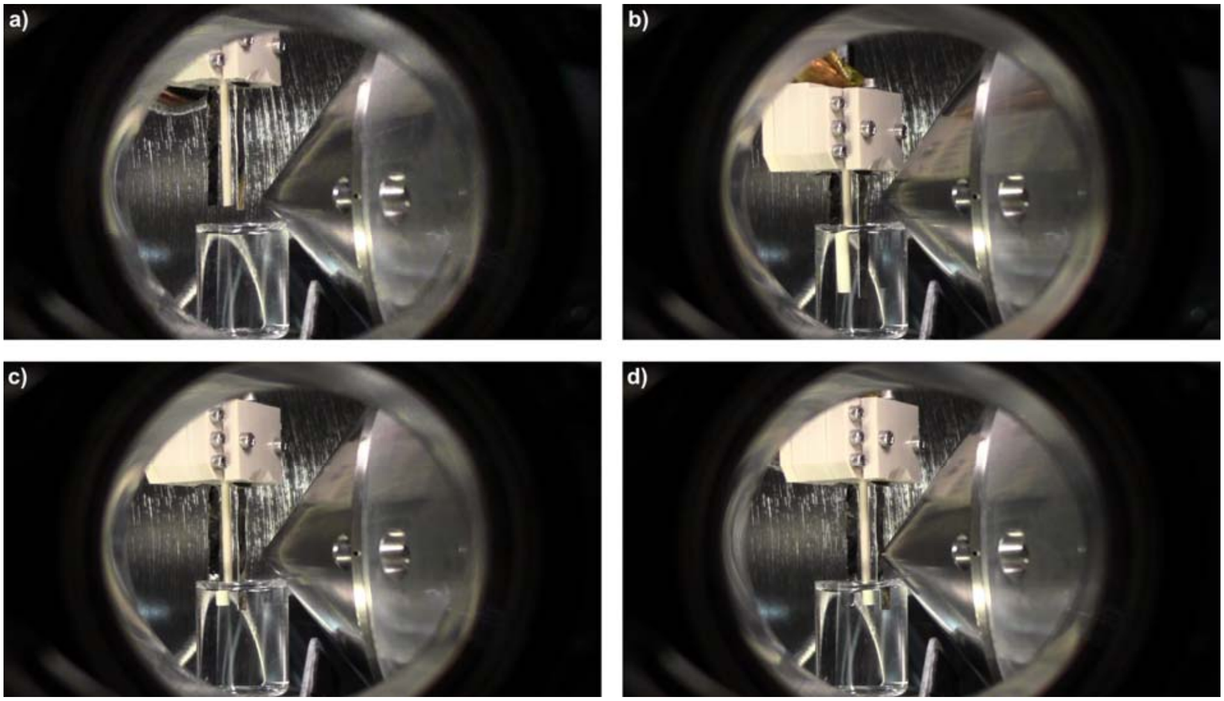

Figure S2. Images of three-electrode electrochemistry setup in the AP-XPS chamber during the "dip & Pull" . (a) Position of electrodes before immersion (b) Electrodes are immersed in the electrolyte, where electrochemical treatment can be performed within the AP-XPS chamber. (c) Electrodes are slowly pulled out of the electrolyte reservoir. (d) Image of the 3-electrode apparatus that has been "dip & pulled" from the electrolyte in the beaker and placed into XPS position while ensuring all three electrodes are in contact with the electrolyte within the beaker.

A video of the "dip & pull" operation can be find at the following link http://www.nature.com/srep

Reference:

(1) Grass, M. E.; Karlsson, P. G.; Aksoy, F.; Lundqvist, M.; Wannberg, B.; Mun, B. S.; Hussain, Z.; Liu, Z. *Review of Scientific Instruments* **2010**, *81*, 053106.

(2) Ogletree, D. F.; Bluhm, H.; Lebedev, G.; Fadley, C. S.; Hussain, Z.; Salmeron, M. *Review of Scientific Instruments* **2002**, *73*, 3872.

(3) Tabata, T.; Shirai, T.; Sataka, M.; Kubo, H. *Atomic Data and Nuclear Data Tables* **2006**, *92*, 375.

(4) Mårtensson, N.; Baltzer, P.; Brühwiler, P. A.; Forsell, J. O.; Nilsson, A.; Stenborg, A.; Wannberg, B. *Journal of Electron Spectroscopy and Related Phenomena* **1994**, *70*, 117.

(5) Matsuda, H.; Daimon, H.; Kato, M.; Kudo, M. *Physical Review E* **2005**, *71*, 066503.

(6) Wannberg, B. *Nuclear Instruments and Methods in Physics Research Section A: Accelerators, Spectrometers, Detectors and Associated Equipment* **2009**, *601*, 182.

(7) Helmer, J. C.; Weichert, N. H. *Applied Physics Letters* **1968**, *13*, 266.

(8) Gelius, U.; Wannberg, B.; Baltzer, P.; Fellner-Feldegg, H.; Carlsson, G.; Johansson, C. G.; Larsson, J.; Münger, P.; Vegerfors, G. *Journal of Electron Spectroscopy and Related Phenomena* **1990**, *52*, 747.

(9) Wannberg, B.; Sköllermo, A. *Journal of Electron Spectroscopy and Related Phenomena* **1977**, *10*, 45.
